# Development and validation of a competitive ELISA based on virus-like particles of serotype *Senecavirus* A to detect serum antibodies

**DOI:** 10.1186/s13568-020-01167-4

**Published:** 2021-01-06

**Authors:** Manyuan Bai, Rui Wang, Shiqi Sun, Yun Zhang, Hu Dong, Huichen Guo

**Affiliations:** 1 State Key Laboratory of Veterinary Etiological Biology and Key Laboratory of Animal Virology of Ministry of Agriculture, Lanzhou Veterinary Research Institute, Chinese Academy of Agricultural Sciences, Xujiaping 1, Lanzhou, 730046 Gansu People’s Republic of China; 2 College of Animal Science, Yangtze University, Jingmi Street, Jingzhou District, Jingzhou, 434025 People’s Republic of China

**Keywords:** *Senecavirus* A, Virus-like particles, Competitive ELISA

## Abstract

Virus-like particles (VLPs) are high-priority antigens with highly ordered repetitive structures, which are similar to natural viral particles. We have developed a competitive enzyme-linked immunosorbent assay (cELISA) for detecting antibodies directed against *Senecavirus* A (SVA). Our assay utilizes SVA VLPs that were expressed and assembled in an *E. coli* expression system as the coating antigens. VLPs have better safety and immunogenicity than intact viral particles or peptides. The VLPs-based cELISA was used to test 342 serum samples collected from different pig farms, and the results showed that its specificity and sensitivity were 100% and 94%, respectively. The consistency rates of cELISA with the BIOSTONE AsurDx™ Senecavirus A (SVA) Antibody Test Kit and an indirect immunofluorescent assay were 90.0% and 94.2%, respectively. Therefore, this VLPs-based cELISA can be effectively and reliably used for the detection and discrimination of SVA infection in serum samples.

## Keypoints

1. We successfully obtained SVA VLPs by using the prokaryotic expression system.
2. We successfully established a SVA antibody-specific competitive ELISA test method by using SVA VLPs as antigens.

## Introduction

SVA, also known as Seneca Valley virus (SVV), is a single-stranded, positive-sense RNA virus. It is the only member of the genus *Senecavirus* in the family *Picornaviridae* (Adams et al. 2015; Hales et al. 2008). SVA has a nonenveloped capsid, approximately 25–30 nm in diameter, with icosahedral symmetry. Its genome consists of a 5′ untranslated region (5′-UTR), an open reading frame (ORF), and a 3′-UTR. The ORF encodes a polyprotein precursor that is cleaved into a leader protein (L) and three proteins (P1, P2, and P3). P1 is processed into VP1, VP3, and VP0, and VP0 is further cleaved into VP2 and VP4, which compose the viral capsid. P2 and P3 are the nonstructural proteins of the virus (Hales et al. 2008; Leme et al. 2016; Liu et al. 2018; Venkataraman et al. 2008).

SVA was initially identified as a contaminant in a culture of adenovirus in human embryonic retinal cells (PER.C6) (Fallaux et al. 1998). It causes vesicular disease in pigs, and its clinical features are very similar to those of foot-and-mouth disease (Canning et al. 2016; Leme et al. 2016; Vannucci et al. 2015). SVA can cause death in piglets (up to 4 days of age), with a mortality rate of 30–70% (Vannucci et al. 2015). In recent years, cases of SVA infection in pigs have been detected in several provinces of China, including Hubei, Fujian, Henan, and Guangdong province (Liu et al. 2018; Qian et al. 2016; Wu et al. 2016). This virus may cause huge economic losses in the pig industry (Knight-Jones and Rushton 2013; Porphyre et al. 2018). Therefore, a rapid, safe, and highly specific diagnostic method is required to prevent and control the spread of SVA.

At present, the serological diagnosis methods for SVA mainly include virus neutralization tests (VNT), indirect immunofluorescent assays (IFAs), competitive enzyme-linked immunosorbent assays (cELISA) and indirect enzyme-linked immunosorbent assays (Dvorak et al. 2017; Leme et al. 2015; Rudin et al. 2011; Yang et al. 2012). VNT is the gold standard for the detection of antibodies in animal sera, and is recommended by the World Organisation for Animal Health (OIE). However, compared with other methods, VNT and IFA are too time-consuming and complex to be suitable for clinical field testing. ELISA is widely used because it is simple, inexpensive, and easy to perform. Inactivated viruses and monomeric proteins are currently used as the coating antigens in ELISA systems for the detection of serum antibodies. However, using complete viral particles as these antigens poses a security risk, and the immunogenicity of monomeric proteins or peptides is relatively poor (Brocchi et al. 1995). Therefore, a recombinant protein that is both safe and highly immunogenic as a diagnostic antigen is critical to the development of new diagnostic techniques (Ko et al. 2009).

VLPs are composed of one or more structural proteins of virus, and do not contain viral genetic material. That structure is similar to native virus particles (Wu et al. 2008). Therefore, VLPs as coating antigens for ELISA systems is safer than that of whole virus particles. Moreover, VLPs exhibit better sensitivity, specificity, and immunogenicity than monomeric proteins or peptides given their highly organized and repetitive surface structures. VLPs have been used to develop safe candidates for immunological detection methods (Michel and Tiollais 2010). In this study, we established a cELISA using SVA VLPs as the coating antigens for the detection of SVA antibody in swine serum samples.

## Materials and methods

### Serum samples and cells

A total of 342 serum samples were harvested from pigs infected with SVA. Antibodies directed against SVA in the serum samples were detected with the commercial BIOSTONE AsurDx™ Senecavirus A (SVA) Antibody Test Kit (South Lake Tahoe, State of California, CA, USA) and an indirect IFA. Serum samples and porcine kidney epithelial cells (IBRS-2) were obtained from the Key Laboratory of the Lanzhou Veterinary Research Institute of the Chinese Academy of Agricultural Sciences, Lanzhou, Gansu Province, China.

### Plasmid construction

pSMK, pSMA and SUMO fusion protein expression vectors, were constructed as described previously (Xiao et al. 2016). The SUMO-tagging recombinant protein expression vectors containing the SVA VP0, VP1 and VP3 gene were constructed as described previously (Mo et al. 2019). In brief, the recombinant plasmids pSMA-VP0, pSMK-VP1 and pSMK-VP3 were constructed using pSMK and pSMA as the expression vector.

### Expression of recombinant proteins

The recombinant plasmids were transferred into *E*. *coli BL21* (DE3). The *E. coli* cells were cultured in LB medium containing ampicillin (50 µg/ml) and kanamycin (10 µg/ml) at 37 ℃. When the OD_600_ was 0.7–0.9, isopropylthio-β-d-galactoside (0.05 mM) was added to LB medium at 16 ℃ to induce coexpression of the recombinant proteins for 16 h.

### Purification of recombinant proteins and quantification of VLPs

The recombinant protein was purified and analyzed with SDS-PAGE and western blotting, as previously described (Mo et al. 2019). The His-SUMO-tag of recombinant protein was cleaved by SUMO protease and the SVA capsid proteins VP0, VP1 and VP3 can self-assemble into VLPs in the assembly buffer (400 mM Tris-HCl, 250 mM NaCl, 1 mM CaCl_2_, and 1 mM DTT, pH = 8.0) for 24 h at 4 °C (Yin et al. 2010). SVA VLPs were purifed by sucrose density gradient centrifugation, the samples were centrifuged at 35,000 rpm for 3 h using an Optima L-100 XP ultracentrifuge (Beckman Coulter, Fullerton, CA, USA). Then the fractions with the highest OD280 value was detected using dynamic light scattering instrument (DLS) and transmission electron microscope (TEM; CM 100, JEOL Ltd., Tokyo, Japan) to determine the molecular size and shape of the SVA VLPs, respectively (Guo et al. 2013).

### Preparation of rabbit serum

Each of three adult rabbits was subcutaneously injected with VLPs (200 µg) and an equal volume of Freund’s complete adjuvant to induce antibody production against the antigen. Two booster immunizations with the same dose of VLPs plus Freund’s incomplete adjuvant were administered at 2-weekly intervals. Two weeks after the final booster injection, the blood of rabbits was collected, and the sera were prepared and stored at − 80 ℃ before analysis.

### Preparation of competitive antibody

The immunoglobulin G (IgG) in the rabbit sera obtained in the previous step was separated with the saturated ammonium sulfate method. The rabbit IgG was further purified with Protein A sepharose affinity column chromatography and then labeled with horseradish peroxidase (HRP) with the improved NaIO_4_ method (Minaeian et al. 2012). The HRP-conjugated rabbit IgG was stored at − 80 ℃ before use.

### Establishment of cELISA method

A 96-well ELISA microplate was coated with various concentrations (0.5–1.0 µg/ml) of SVA VLPs in carbonate buffer solution (0.05 M, pH 9.6) and incubated at 4 ℃ overnight. After the microplate was washed three times with phosphate-buffered saline (PBS) containing 0.1% Tween (PBST), it was blocked with 1% bovine serum albumin (BSA) in distilled water for 1 h at 37 ℃, washed three times with PBST, and patted dry. In the antibody-coated wells of the plate, 50 µl of SVA-positive or SVA-negative serum and 50 µl of serially diluted HRP–IgG were mixed to ensure that the competitive reaction was as efficient as possible. The plates were incubated for 60 min at 37 ℃ and washed 3–4 times with 300 µl of PBST.

Tetramethylbenzidine (TMB, 50 µl) was added and the samples incubated for 15 min at 37 ℃. The color reaction was stopped with 50 µl of 2 M H_2_SO_4_. To optimize the reaction conditions of the SVA cELISA, the best results under each set of condition were determined on the basis of the OD_450_ values and the percentage inhibition (PI), which was calculated with the following formula: PI = (OD_450_ of standard negative serum – OD_450_ of measured individual sample)/(OD_450_ of standard negative serum – OD_450_ of standard positive serum) × 100%.

### Determination of the cELISA cut-off value (PI)

In this procedure, 50 SVA-positive seras with different antibody titers and 50 SVA-negative sera were tested by the established cELISA to determine the PI. The cut-off value was determined based on a receiver operating characteristic (ROC) curve.

### Analysis of the sensitivity and specificity of the cELISA

The sensitivity of the cELISA was assessed with SVA-positive sera with different antibody titers. To determine whether the SVA cELISA system reacted positively with antibodies directed against viruses other than SVA, six randomly selected pig sera positive for serotype O FMDV, PCV2, PPV, CSFV, *Actinobacillus pleuropheumoniae*, or *Haemophilus parasuis* were tested with the SVA cELISA.

### cELISA repeatability test

A repeatability test was conducted to determine the stability of the SVA cELISA. The intra-assay repeatability of the SVA cELISA was tested with 10 randomly selected serum samples, with three replicates of each, in the same microplate. The inter-assay repeatability of the SVA cELISA was used to test the serum samples in triplicate in microplates in different production batches. The coefficients of variation (CVs) of the three replicates of each of the 10 serum samples were calculated.

### Comparison of cELISA and commercial ELISA

To validate the SVA cELISA, a total of 342 swine sera samples were tested with both the SVA cELISA and the BIOSTONE AsurDx™ Senecavirus A (SVA) Antibody Test Kit.

### Comparison of cELISA and IFA

When cells dense were about 60% in cell culture flasks, they were infected with the virus, and the virus solution was discarded at the appropriate time. The cells were then washed three times with PBS, fixed with 4% paraformaldehyde for 10–15 min, and washed three times with PBS. The cells were treated with 0.1% Triton X-100 for 15 min and washed three times with PBS. An anti-SVA polyclonal antibody was added, and the cells were incubated at 37 ℃ for 2 h. The cells were washed three times with PBS, FITC-labeled anti-porcine IgG was added, and the cells were incubated for a further 1 h at 37 ℃ in an incubator. The cells were washed three times with PBS and observed under an inverted fluorescence microscope.

## Results

### Expression and identification of recombinant VP0, VP1 and VP3 proteins

**Fig. 1:**
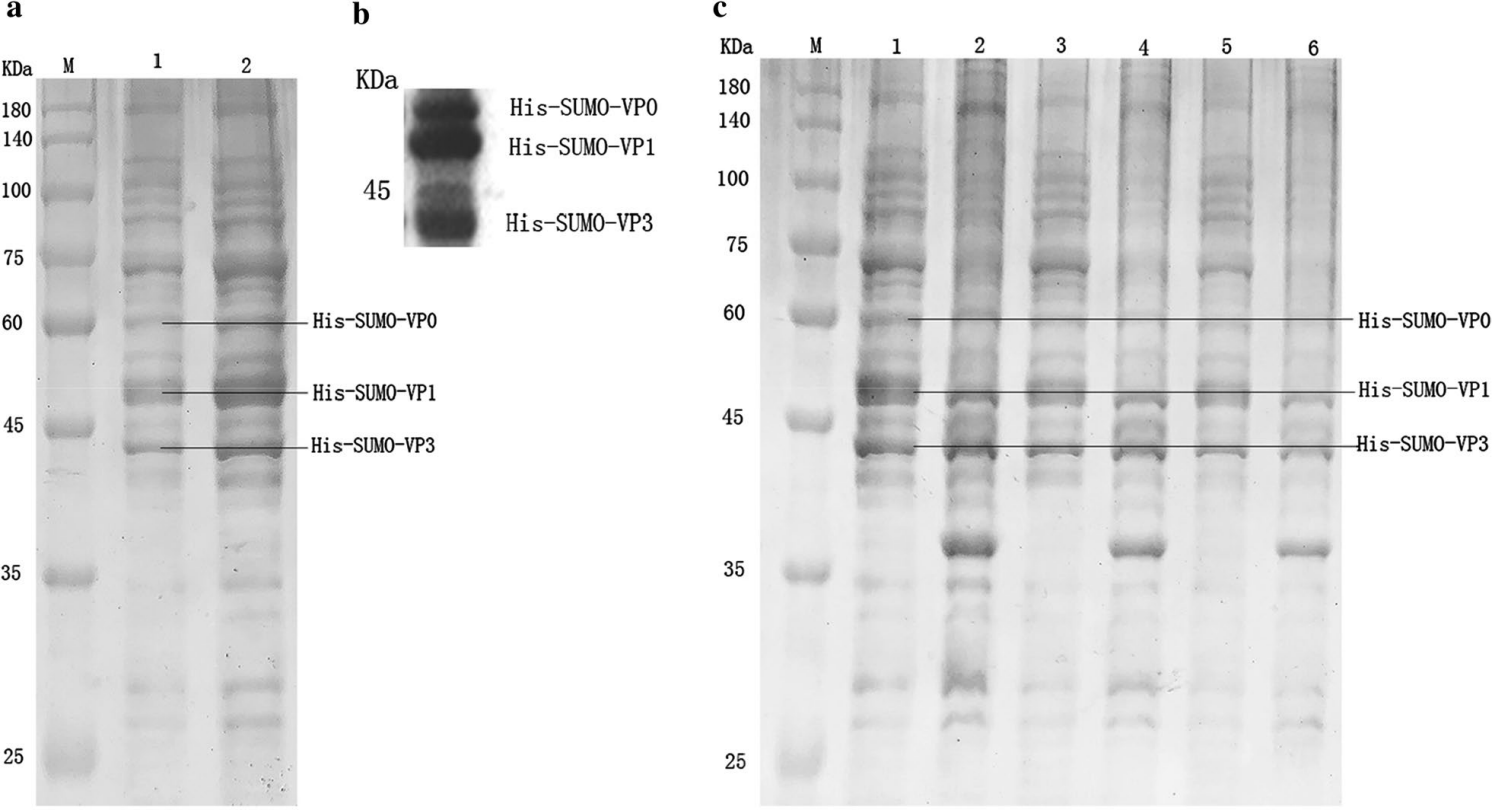
Analysis of three SUMO-tagged recombinant proteins using SDS-PAGE and western blotting (**a**). SDS-PAGE: M, protein molecular marker; lane 1, prior to induction; lane 2, post induction. The SUMO-tagged recombinant proteins were induced as described in "Materials and methods". Identification of recombinant proteins with varied preparations using western blot (**b**). Lanes 1, recombinant proteins generated in varied preparations. Expression of recombinant proteins induced at 20 °C and 37 °C (**c**). Protein molecular marker (M), supernatant (lane 1) and pellet (lane 2) of lysated recombinant *E. coli* incubated at 20 °C, supernatant (lane 3) and pellet (lane 4) of lysated recombinant *E. coli* incubated at 37 °C; and supernatant (lane 5) and pellet (lane 6) of lysated recombinant *E. coli* prior to induction

The recombinant fusion proteins (VP0, VP1, and VP3) were purified with nickel-chelating affinity chromatography. The results of SDS-PAGE indicated that the recombinant fusion proteins were successfully expressed (Fig. 1a), and the results of a western blotting analysis indicated that the recombinant fusion proteins were recognized by standard SVA-positive serum (Fig. 1b). The yields of the soluble recombinant proteins were greater when the expression was induced at 20 ℃ than when it was incubated at 37 ℃ (Fig. 1c), which is consistent with previous findings (Mo et al. 2019).

### Assembly of SVA VLPs

**Fig. 2:**
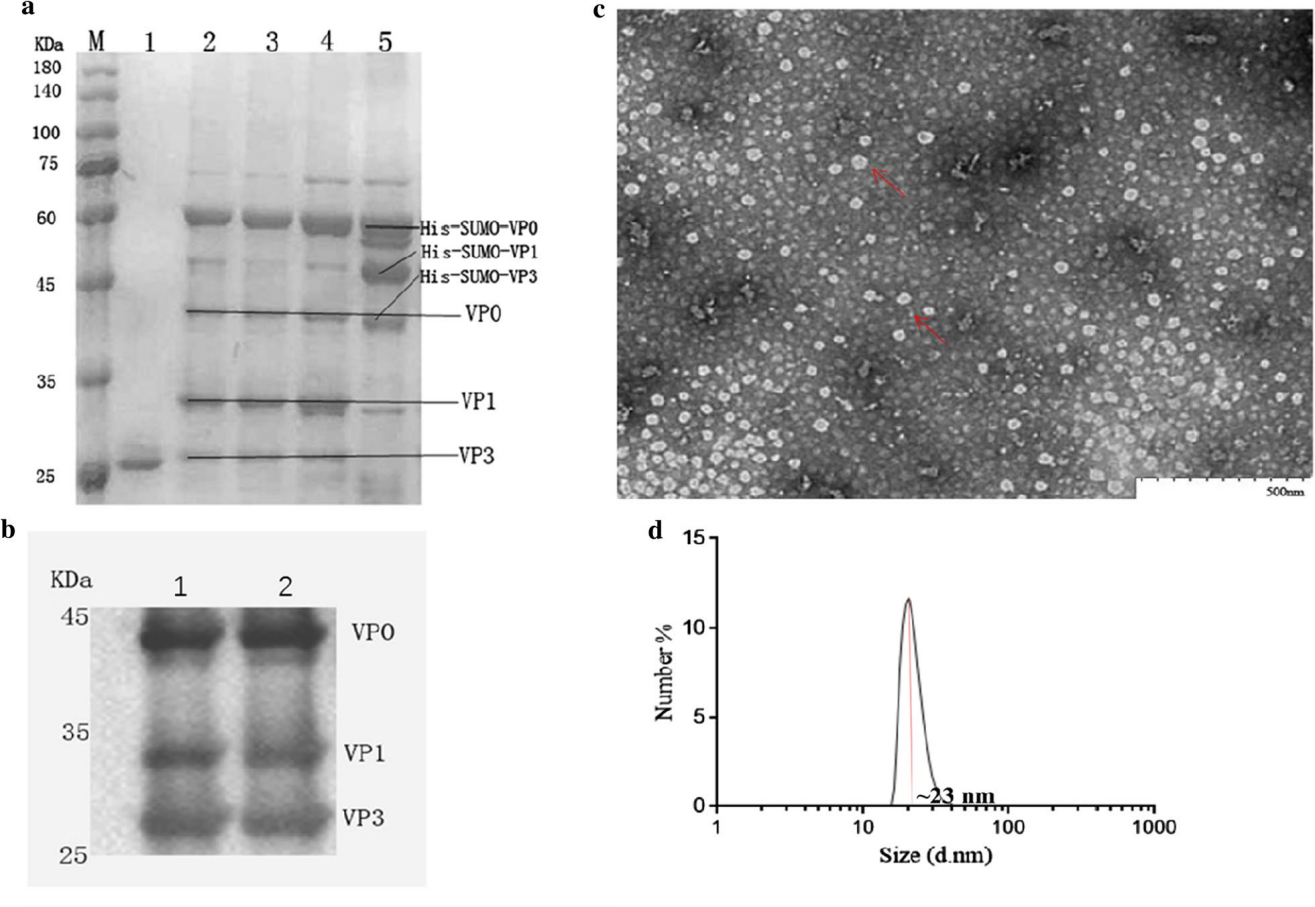
Expression and assembly of purified SVA-VLPs (**a**). Analysis of SVA virus-like particles using SDS-PAGE. M, protein molecular marker; lane1, SUMO Protease; lane 2–4, digested recombinant proteins with SUMO protease; lane 5, nondigested recombinant proteins. Analysis of SVA virus-like particles using western blot (**b**). Lanes 1–2, purified SVA-VLPs generated in varied preparations. Visualization of SVA virus-like particles using TEM (**c**). The bar indicates 200 nm. DLS diagram (**d**)

After the assembly of the SVA VLPs in assembly buffer, the product was filtered, and the immunogenicity of the SVA VLPs was confirmed with SDS-PAGE (Fig. 2a) and western blotting (Fig. 2b). The molecular size and shape of the SVA VLPs was determined with TEM (Fig. 2c) and DLS (Fig. 2d), the results show that the diameter of SVA VLPs was ~ 23 nm, which was similar to natural virions of SVA.

### Preparation of rabbit HRP-conjugated hyperimmune serum

A direct ELISA was used to determine the titer of HRP–IgG, and showed it to be 1:512,000 (Table 1).

**Table 1:** ELISA results of HRP conjugated antibody

	Diluted from 10 mg/ml	Antibody concentration (ng/ml)	HRP conjugated antibody
1	1:1000	10000.00	2.716
2	1:2000	5000.00	2.286
3	1:4000	2500.00	1.578
4	1:8000	1250.00	1.045
5	1:16,000	625.00	0.678
6	1:32,000	312.50	0.443
7	1:64,000	156.25	0.266
8	1:128,000	78.13	0.194
9	1:256,000	39.07	0.129
10	1:512,000	19.54	0.117
11	Blank	Blank	0.053
12	Blank	Blank	0.053
	Titer	Titer	1:512,000

### Development of cELISA based on SVA VLPs

The optimal reaction conditions for the SVA cELISA were confirmed with OD_450_ and P/N (positive/negative) values: 0.9 µg/ml of VLPs in 100 µl volume (carbonate solution) were used to coat each well of the plate at 4 ℃ overnight; the plate was blocked with 1% BSA for 75 min at 37 ℃; the competitive reaction was performed for 30 min at 37 ℃; a 1:3 dilution of the test serum sample and a 1:7000 dilution of HRP–IgG were used; reaction with TMB substrate for 15 min at 37 ℃ to visualize the products; the reaction was terminated with 50 µl of 2 M H_2_SO_4_.

### Determination of the cELISA cut-off value (PI)

**Fig. 3:**
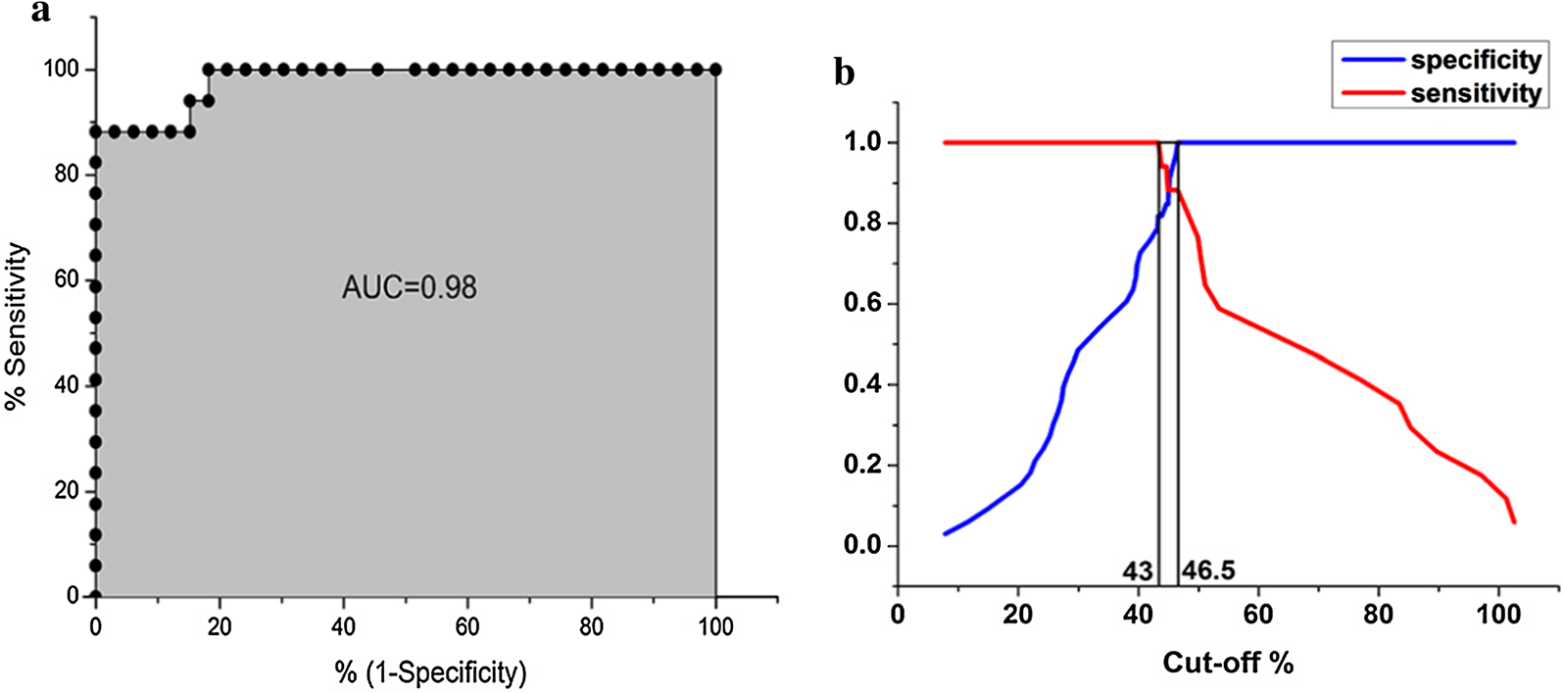
Validation of cELISA by ROC curve (**a**). The PI values of 104 confirmed sera calculated by cELISA were compared with the virus neutralization test results via ROC analysis. AUC stands for area under curve. The value of sensitivity or specificity of the cELISA calculated at varied cutoff value (**b**)

A total of 50 positive sera and 50 negative sera were tested with the SVA VLP cELISA. The PI cut-off point was confirmed that minimized the false negative and false positive rates of the SVA cELISA system (Fig. 3a, b). A ROC curve analysis showed that the specificity and sensitivity of the cELISA were optimal when the cut-off value was PI = 45%. Therefore, serum samples were defined as positive when the PI of the serum tested was ≥ 45%, and negative when PI < 45%.

### Evaluation of the cELISA

A total of 342 swine serum samples were tested using the SVA VLP cELISA, the BIOSTONE AsurDx™ Senecavirus A (SVA) Antibody Test Kit, and IFA. The calculated sensitivity and specificity of the SVA cELISA were 94% and 100%, respectively. The coincidence rates when the cELISA was compared with the commercial AsurDx™ Senecavirus A (SVA) Antibody Test Kit Manual and IFA were 90% and 94.2%, respectively (Table 2). These results confirm that the SVA cELISA is highly accurate. The repeatability of the assay was evaluated by determining the OD_450_ of 10 serum samples processed in triplicate in the same plate (intra-assay repeatability) or different plates at different times (inter-assay reproducibility). The standard deviations and CVs were then calculated. The low values of CV achieved in the repeatability (CV < 10%) and reproducibility (CV < 15%) assays demonstrate the reliability of this new approach (Tables 3 and 4).

**Table 2:** Comparison of the competitive ELISA with commercial kit for AsurDxTM Senecavirus A (SVA) Antibody Test Kit and IFA

	cELISA		BIOSTONE AsurDxTM	IFA
	Positive	Negative	Total	Total
BIOSTONE AsurDxTM				
Positive	220	16	236	
Negative	18	88	106	
IFA				
Positive	90	1		91
Negative	6	23		29
CELISA				
Total	238	104	342	120

**Table 3:** Intra-batch reproducibility test of VLPs-cELISA

Sample ID	1	2	3	X^a^	SD^b^	CV%^c^
#1	73.43	79.27	72.66	75.12	2.95	3.93
#2	35.93	39.97	40.80	38.90	2.13	5.47
#3	71.57	81.52	82.71	78.60	4.99	6.35
#4	17.41	19.87	18.34	18.54	1.02	5.48
#5	23.15	24.65	26.88	24.89	1.53	6.16
#6	45.83	45.47	54.06	48.45	3.96	8.18
#7	48.61	41.42	53.56	47.86	4.99	10.42
#8	37.04	30.78	34.95	34.26	2.60	7.59
#9	43.61	36.19	39.73	39.84	3.03	7.61
#10	25.74	24.47	27.05	25.75	1.05	4.09

^a^Mean PI value

^b^Standard deviation

^c^Coefficient of variation

**Table 4:** Inter-batch reproducibility test of VLPs-ELISA

Sample ID	1	2	3	X^a^	SD^b^	CV%^c^
#1	68.87	68.59	61.29	66.25	4.30	6.49
#2	27.17	27.76	31.01	28.65	2.07	7.22
#3	71.93	93.80	88.34	84.69	11.38	13.44
#4	19.62	16.65	16.74	17.67	1.69	9.55
#5	49.76	58.69	48.55	52.33	5.54	10.58
#6	45.06	40.88	39.62	41.85	2.84	6.79
#7	33.96	30.64	29.49	31.36	2.32	7.40
#8	29.72	30.59	27.63	29.31	1.52	5.18
#9	48.30	49.17	54.05	50.51	3.10	6.14
#10	9.89	10.13	8.00	9.34	1.17	12.47

^a^Mean PI value

^b^Standard deviation

^c^Coefficient of variation

## Discussion

Since 2015, SVA has spread increasingly widely, and the symptoms of SVA infection are similar to those of *foot-and-mouth disease*, which has caused great economic losses in the pig industry. Therefore, the rapid diagnosis of SVA infection is crucial to its prevention and control (Montiel et al. 2016). We have developed a sensitive and specific SVA ELISA for the detection and diagnosis of SVA-specific antibodies. Previous studies have shown that the immunization of animals with FMDV VLPs produced in *E. coli* was sufficient to induce antibodies that protected guinea pigs, pigs, and cattle from virulent infection, indicating the potential utility of VLPs as candidate vaccines (Guo et al. 2013). The *E. coli* expression system can be easily used to manufacture VLPs and does not require cell culture or bio-protection facilities. Our previous studies and those of others have confirmed the immunogenicity of VLPs (Guo et al. 2013; Li et al. 2016; Li et al. 2011). However, the reactivity of VLPs as coating antigens for cELISA remained unclear. Therefore, we developed a cELISA based on SVA VLPs produced in an *E. coli* expression system. Therefore, we developed a cELISA method based on the SVA VLP generated in the *E. coli* expression system, and tested the serum samples. The result demonstrated that it has high specificity, sensitivity, and repeatability. In this experiment, the SVA capsid protein genes were inserted into the pSMK or pSMA plasmids with His and SUMO (small ubiquitin-modified system) tags, to generate pSMA-VP0, pSMK-VP1, and pSMK-VP3. Competent *E. coli* cells were cotransformed with the plasmids and the coexpression of the capsid proteins was induced. In this experiment, the animals were immunized with the recombinant proteins to obtain highly immune serum. Therefore, the basic components of the diagnostic test, including the diagnostic antigens and related reference sera, can be generated independently. At present, the development of diagnostic reagents based on inactivated SVA requires complex biosafety equipment, increasing its cost. For example, in the BIOSTONE AsurDx™ Senecavirus A (SVA) Antibody Test Kit (BIOSTONE, USA), inactivated SVA and a monoclonal antibody are used to detect serum antibodies, and the kit can test 450 sera samples. However, it costs $1500, and is consequently too expensive for developing countries. Therefore, we have established a bacterial expression system for SVA VLPs, which can be prepared inexpensively in any laboratory with a device for bacterial culture. In this assay, SVA VLPs replace inactivated SVA in the ELISA, so that the cELISA can be used to assess clinical herd immunity, particularly in developing countries.

In some cases, different monoclonal antibodies (mAbs) should be used in combination to achieve the optimal performance of an ELISA and to overcome the weak affinity of serum antibodies for the coating antigen. Although mAbs may show high specificity, high sensitivity, and consistent performance, their use will increase the complexity and uncertainty of the test method (Yang et al. 2015). In the present study, polyclonal antibodies were used as the competitive antibodies to evaluate the level of clinical herd immunity. Polyclonal antibodies have greater affinity for the antigen because they recognize more epitopes on the coating antigen than mAbs, which represented high specificity and good performance.

In summary, this cELISA based on SVA VLPs expressed in an *E. coli* system has high specificity, sensitivity, and reproducibility. The SVA VLPs cELISA does not require high-level biosafety facilities, is easy to produce, and inexpensive, so it is especially suitable for use in underdeveloped countries. And it can be used to assess herd immunity induced with a variety of inactivated SVA vaccines, promoting the prevention and control of SVA.

## Data Availability

Accession number, DQ641257.1 identified the nucleic acid sequence of the SVA. All materials are available upon request.
